# Authors’ reply

**DOI:** 10.4103/0970-2113.80346

**Published:** 2011

**Authors:** Arunabha Datta Chaudhuri, Sourin Bhuniya, Sudipta Pandit, Atin Dey, Subhasis Mukherjee, Pulakesh Bhanja

**Affiliations:** *Department of Chest Medicine, R. G. Kar Medical College, Kolkata, India*; 1 *Department of Chest Medicine, Calcutta National Medical College, Kolkata, India. E-mail: arunabhadc@rediffmail.com*

Sir,

We would like to thank readers for showing interest in our article[1] and expressing views on this topic.[2] We do agree that PCR gives more rapid results and improved sensitivity and specificity than sputum smear for AFB in the diagnosis of tuberculous pleural effusion. However, the sensitivity of the nucleic acid amplification (NAA) technique depends on many factors like the number of mycobacteria, their homogenous distribution in the sample, the presence of amplification inhibitors, the type of primer used and the genomic sequence amplified.[3] Contamination either with extraneous DNA or with amplification products of previous analysis or the presence of nonviable organisms is the principal source of false-positive results. There is higher frequency of PPD positivity (which is indicative of prior infective exposure to *Mycobacterium tuberculosis*) among patients with PCR positivity, compared to those with PCR negativity, thereby implying that even latent tubercular infection can give rise to positive PCR results.[4]

A pooled analysis of the data from 20 studies assessing the use of pleural fluid NAA tests concluded that these tests demonstrated reasonably high specificity (97% for commercial and 91% for in-house tests), but generally poor and variable sensitivity (62% for commercial and 76.5% for in-house tests).[5] An earlier meta-analysis of 40 studies came to a very similar conclusion.[6] The disappointingly low sensitivities of NAA techniques might be due to the presence of inhibitors in the pleural fluid or to intracellular sequestration of the mycobacteria.

Therefore, the disadvantages of PCR including high cost, risk of contamination and the technology involved in the procedure do not permit its routine diagnostic use at present.
